# Long-Lasting Production of New T and B Cells and T-Cell Repertoire Diversity in Patients with Primary Immunodeficiency Who Had Undergone Stem Cell Transplantation: A Single-Centre Experience

**DOI:** 10.1155/2014/240453

**Published:** 2014-12-01

**Authors:** Monica Valotti, Alessandra Sottini, Arnalda Lanfranchi, Federica Bolda, Federico Serana, Diego Bertoli, Viviana Giustini, Marion Vaglio Tessitore, Luigi Caimi, Luisa Imberti

**Affiliations:** ^1^CREA, Diagnostics Department, Spedali Civili of Brescia, 25123 Brescia, Italy; ^2^Department of Pediatric Oncohaematology and Bone Marrow Transplantation, Spedali Civili of Brescia, 25123 Brescia, Italy

## Abstract

Levels of Kappa-deleting recombination excision circles (KRECs), T-cell receptor excision circles (TRECs), and T-cell repertoire diversity were evaluated in 1038 samples of 124 children with primary immunodeficiency, of whom 102 (54 with severe combined immunodeficiency and 48 with other types of immunodeficiency) underwent hematopoietic stem cell transplantation. Twenty-two not transplanted patients with primary immunodeficiency were used as controls. Only data of patients from whom at least five samples were sent to the clinical laboratory for routine monitoring of lymphocyte reconstitutions were included in the analysis. The mean time of the follow-up was 8 years. The long-lasting posttransplantation kinetics of KREC and TREC production occurred similarly in patients with severe combined immunodeficiency and with other types of immunodeficiency and, in both groups, the T-cell reconstitution was more efficient than in nontransplanted children. Although thymic output decreased in older transplanted patients, the degree of T-cell repertoire diversity, after an initial increase, remained stable during the observation period. However, the presence of graft-versus-host disease and ablative conditioning seemed to play a role in the time-related shaping of T-cell repertoire. Overall, our data suggest that long-term B- and T-cell reconstitution was equally achieved in children with severe combined immunodeficiency and with other types of primary immunodeficiency.

## 1. Introduction

Although primary immunodeficiencies (PID) are considered rare, the number of patients with these diseases has increased in recent years, and about 150 different forms of PID have been identified [1]. Despite the variability of PID etiology, hematopoietic stem cell transplantation (HSCT) remains the major curative treatment to correct the immunodeficiency and reverse a poor prognosis, especially for severe combined immunodeficiency (SCID) in which, however, also gene therapy has successfully been used [2, 3]. Because SCID is a genetic disease, less than 25% of these children will have a healthy, HLA-matched sibling donor available [4]. Therefore, grafting of HLA-haploidentical parental cells is the treatment of choice for these patients, and it is being employed with increasing success [5]. A feasible alternative source of stem cells is cord blood bank donors, as well as mismatched, related blood donors; HSCT from these sources have similar outcomes [4].

Assessment of the early lymphocyte reconstitution is becoming a key issue because posttransplantation infections may be directly related to incomplete recovery. Indeed, immune reconstitution, which refers to quantitative immune cell repopulation, does not necessarily associate with immune recovery, which requires a qualitative restitution.

Several factors affect the immune recovery, including age, sex, initial pathology, conditioning regimen, degree of genetic differences between donor and recipient, source of stem cells, and post-HSCT events, such as acute and chronic GvHD and infectious complications [6]. The increase of naive T-cell count and the enlargement of T-cell diversity occur in most patients with SCID during the first year after HSCT [7, 8]. The increase in T cells containing T-cell receptor (TCR) excision circles (TRECs) early after HSCT appears to be the most predictive marker for a long-lasting T-cell reconstitution [9], while a restitution of T-cell function occurring in the first 6 months has been associated with improved survival [8].

Much less information is available regarding the level of TRECs and the extent of TCR diversity, which is strictly dependent on the contribution of newly produced T cells [10], in subjects who underwent HSCT for SCID a long time before. Analogously, no data are available on levels of K-deleting recombination excision circles (KRECs), which are considered a reliable marker of bone marrow output [11, 12].

Therefore, in this retrospective study we examined the long-term lymphocyte reconstitution by analysing the levels of KRECs, TRECs, and T-cell diversity in 54 SCID patients who had undergone HSCT up to 22 years before, and we compared the results with those of 48 patients with other types of PID, who also received HSCT and with those of 22 patients with PID that had not been subjected to transplantation.

## 2. Patients and Methods

### 2.1. Patients

The retrospective analysis was performed in 1038 samples collected from April 1999 to March 2014. Samples belonged to 124 children whose diagnosis was based on clinical findings, family history, and laboratory evaluation of immune function. Genetic confirmation of the disease was obtained for the majority of patients. Of the 102 children that received allogeneic HSCT at the Department of Pediatrics, Spedali Civili of Brescia, Italy, 54 had a T^−^B^+^ or T^−^B^−^ SCID (“HSCT-SCID” group) and 48 were affected by other PID (“HSCT-PID” group). The remaining 22 children were not subjected to HSCT (“no-HSCT” group). Only patients of whom at least five consecutive samples were available were included in the study.

HSCT was performed from a HLA-matched sibling donor or from an unrelated donor, identified by registry search via the National Marrow Donor Program (http://ibmdr.galliera.it/), or from mismatched related family donors. The method for T-cell depletion (monoclonal antibody depletion or CD34^+^ selection) depended on the year in which HSCT was performed. Conditioning regimen followed the guidelines of the European Bone Marrow Transplantation/European Society for Immune Deficiency in use at the time of transplantation.

The study was reviewed and approved by the Hospital Ethics Boards (protocol n. 1417 of June 26, 2013).

### 2.2. Laboratory Evaluations

Chimerism analysis was performed by DNA analysis at highly polymorphic loci (D1S80, HLA-DQa, and ApoB) and, since 2006, it had been assessed on positively selected CD3^+^ and CD19^+^ lymphocytes [13].

The evaluation of TRECs in the samples analysed before 2010 was done as described by Zhang et al. [14], while in those obtained later was performed by a duplex quantitative real-time PCR (as described by Sottini et al. [11]), which was also used for the quantification of KRECs. Copy numbers of KRECs, of TRECs, and of a segment of TCR alpha constant gene, which is used as the reference gene, were obtained by interpolating the standard curve, which is obtained by serial dilutions of a linearized plasmid DNA, containing three inserts corresponding to fragments of KRECs, TRECs, and reference gene.

Because KREC and TREC production is age-related [11] and two different methods had been used for the quantification of TRECs, six distinct cut-offs were calculated that correspond to the 5th percentiles [11, 15] of the values obtained in 236 healthy children, who were divided into those younger and older than 3 years (<3 or >3 years old). The cut-offs for KRECs were 46535 KRECs/mL in children <3 years and 5841/mL in those >3 years; the cut-offs for TRECs evaluated with the older method were 22350/10^6^ cells for children <3 years and 13284/10^6^ cells for those >3 years; the cut-offs for TRECs obtained with the newer method were 30128 TRECs/mL for those <3 years and 13369 for those >3 years. The above reported values are very similar also to those obtained when KRECs and TRECs were measured in whole blood spotted on Guthrie card [16].

TCR beta variable genes (TCRBV) were amplified, after RNA isolation and cDNA synthesis, using TCRBV-specific primers; then PCR products were subjected to heteroduplex analysis [17]. In this assay, denatured and renatured amplified products of TCRBV migrate in polyacrylamide gels as smears in the case of a polyclonal repertoire, whereas heteroduplex or homoduplex bands indicate oligoclonal or clonal conditions. The performance of this method was periodically verified by simultaneous CDR3 spectratyping analysis [18]. T-cell repertoire was considered unrestricted if polyclonal smears were seen in all lanes of heteroduplex gel or if oligoclonal bands were found in 1 to a maximum of 4 lanes.

### 2.3. Statistical Analysis

At any time point, a normal or insufficient KREC and TREC release (calculated starting from the time of HSCT in HSCT-SCID and HSCT-PID, and since birth in no-HSCT) were defined by two binary variables representing, respectively, samples being over or under the cut-offs of KRECs and TRECs. For a convenient descriptive analysis of data (and not for the statistical inference), time variables were grouped into seven classes. When a patient had more than one sample falling into the same time-class, a proportion representing the average number of samples over or under the cut-off within that time-class was calculated. Then, the between-patient means of all these average proportions were calculated and plotted as bar charts. Therefore, in the plots each bar represents a mean “adjusted” frequency of patients with KRECs and TRECs above the appropriate cut-off for that age, therefore having a “normal” bone marrow or thymic release of new lymphocytes. The same procedure was employed to represent the mean “adjusted” frequency of patients with an unrestricted repertoire. To assess the statistical significance of these frequency changes during the follow-up, the longitudinal analysis of the probability of being over or under the cut-off was estimated by logistic regression using mixed models with a random intercept. In this case, time was considered as continuous, and therefore each individual observation could be used without “averaging” over the time-classes. To properly model the observed convex shape of the probability variation of these immune parameters during the follow-up, time was also inserted into every model as a quadratic term. Model selection was performed with a forward stepwise procedure, and a likelihood ratio test was used for model comparisons. Linear contrasts were performed to test* a priori* hypothesis. Statistical significance was set at *P* < 0.05.

## 3. Results

### 3.1. Patients

The type of immunodeficiency, classified according to Al-Herz et al. [19], and the mutations causing the diseases, if known, are summarized in Table 1, which also reports the age at the time of HSCT, type of HSCT, conditioning regimen, occurrence and type of GvHD, serotherapy, and length of the follow-up.

The number of samples analysed for each patient, the number of samples belonging to transplanted and nontransplanted patients, age-classes at the time of sampling, patients' age at the first and last sampling, and the years of post-HSCT follow-up were described in Supplementary Figure 1 (see Supplementary Material available online at http://dx.doi.org/10.1155/2014/240453). The post-HSCT follow-up period was fewer than 5 years for 8 children, but most of them were followed up for 8–10 years.

### 3.2. Evaluation of KREC and TREC Levels

**Figure 1:**
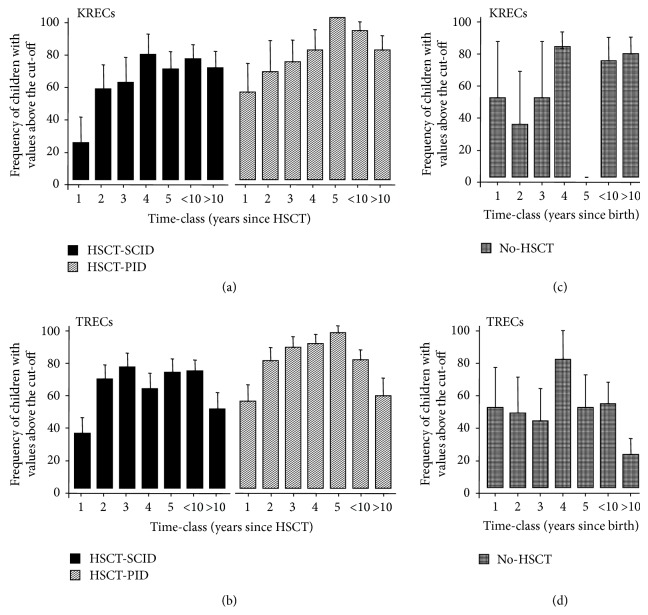
((a) and (b)) Average frequency of children with SCID and PID receiving HSCT (HSCT-SCID and HSCT-PID, resp.) with values of KRECs and TRECs over the respective cut-offs, calculated in age-matched healthy children. ((c) and (d)) Average frequency of children with SCID and PID that were not subjects to HSCT (no-HSCT), with values of KRECs and TRECs over the cut-offs. The indicated time-classes for HSCT children were calculated starting from the date of HSCT, whereas in no-HSCT children each time-class represents the age at the time of sampling. Time-classes were indicated as follows: 1: <1 year, 2: <2 years, 3: <3 years, 4: <4 years, 5: <5 years, <10: <10 years, and >10: >10 years. Bars represent the means and error bars represent the standard deviations.

Samples of transplanted and nontransplanted patients were retrospectively evaluated by using binary variables that classified children as having KRECs and TRECs under or above the cut-off appropriate for the children's age and, in the case of TRECs, also for the method employed at the moment of blood sampling. In children with SCID the mean adjusted frequency of patients with KRECs above the cut-off was 23% in the first year after HSCT, progressively increased to 77% at 4 years, and then slightly decreased but remained 69% also at more than 10 years since transplantation (Figure 1(a)). A similar trend was observed in transplanted children with other PID, even if the percentages were always higher, with almost all children having “normal” (above the cut-off) KRECs at 5 years since HSCT. At 10 years or more, 80% of children still showed normal values of KRECs (Figure 1(a)).

The mean adjusted percentage of SCID children with levels of TRECs above the cut-off was 34% 1 year after HSCT, steeply increased to 68% at the following time point, and remained between 60% and 80% until 10 years after HSCT, when it decreased to a mean of 49% (Figure 1(b)). In HSCT-PID group, the mean adjusted frequency of TRECs above the cut-off was 53% in the first year; then the pattern of increase was similar to that of KRECs, with higher values at 5 years, and a quick decrease to the level observed at the first time-class at more than 10 years after transplantation (Figure 1(b)). Repeated-measure logistic models estimating factors affecting the probability of being above the cut-offs confirmed the increasing-decreasing shape of these time-trends (odds ratio <1 for the quadratic term of the regression) and indicated that the average frequency of patients with normal KREC and TREC values was not different in HSCT-SCID and HSCT-PID children (Table 2 and Supplementary Figure 2).

The levels of KRECs and TRECs were also evaluated in the longitudinal samples of no-HSCT children in order to investigate the kinetics of KREC and TREC release in an appropriate control group. In this case, to employ a common time scale, patients' age at the time of sampling was used (Figures 1(c) and 1(d)). The mean adjusted frequency of patients with KRECs above the cut-off remained constantly high in older patients (in the 5-year-old age-class the mean is 0% because only one child fell into this class and he had KRECs under the cut-off); on the contrary, the mean adjusted frequency of patients with TRECs over the cut-off, which was about 80% when these children were 4 years old, decreased more deeply than in the HSCT groups to become 21% in children older than 10 years. Logistic regression confirmed that these opposite patterns of KREC and TREC reconstitution in no-HSCT groups were significantly different from those of HSCT groups (Table 3 and Supplementary Figure 3).

### 3.3. T-Cell Repertoire Analysis

**Figure 2:**
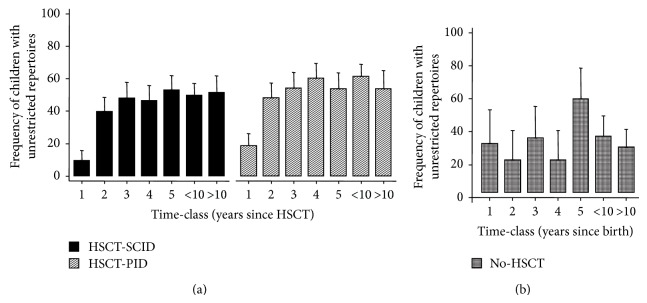
((a) and (b)) Average frequency of children with SCID and PID receiving HSCT (HSCT-SCID and HSCT-PID, resp.) or that were not subjects to HSCT (no-HSCT) that show unrestricted T-cell repertoires. The indicated time-classes for HSCT children were calculated starting from the date of HSCT, whereas in no-HSCT children each time-class represents the age at the time of sampling. Time-classes were indicated as follows: 1: <1 year, 2: <2 years, 3: <3 years, 4: <4 years, 5: <5 years, <10: <10 years, and >10: >10 years. Bars represent the means and error bars represent the standard deviations.

The mean adjusted frequency of patients having unrestricted T-cell repertoires was only 10% in HSCT-SCID patients at 1 year after transplantation, increased to 40% in the following year, and remained between 40% and 55% in the remaining observation time. Therefore, HSCT-SCID children, in spite of the decreasing mean frequency of normal TREC levels observed at the last two time points of the follow-up, maintained the acquired T-cell heterogeneity. In HSCT-PID patients, the modulation of T-cell repertoire was very similar to that of the previous group (Figure 2(a)), and this was confirmed by a logistic regression analysis showing that the pattern of change over time of the probability of having an unrestricted repertoire did not differ between the two groups (Table 2 and Supplementary Figure 2). In no-HSCT patients the frequency of patients with an unrestricted repertoire was below 40% in all time-classes, except in 5-year-old patients, in whom it reached 57% (Figure 2(b)). This significantly lower frequency of patients with an unrestricted repertoire among the no-HSCT patients was also demonstrated by logistic regression (Table 3, Supplementary Figure 3).

### 3.4. Analysis of Factors That Potentially Influence KREC and TREC Levels and T-Cell Repertoire

By multivariable logistic regression we investigated whether factors, such as the source of stem cells (from HLA-matched sibling, mismatched related family, or unrelated donors), the dose of stem cells, the presence and degree of T-cell depletion, the type of conditioning, the presence of GvHD at any time during the follow-up, the presence of a complete B- or T-cell engraftment, and the age at the time of transplantation, had an influence on the probability of having “normal” KRECs, TRECs, or T-cell repertoire. Using these factors, we could not build models fitting the data sufficiently well, excluding those reported in Table 4 describing factors affecting the probability of developing an unrestricted T-cell repertoire over time, which were as follows: T-cell reconstitution, time since transplantation, GvHD, and ablative conditioning. According to the model, the probability of having a recovered, unrestricted T-cell diversity during the follow-up is strongly associated with a normal production of TRECs (odds ratio: 3.239, *P* < 0.001) and is different between patients presenting with or without GvHD, particularly those not having undergone an ablative conditioning. Indeed, the models predict a peculiar pattern during time (Supplementary Figure 4): patients with GvHD have a slower recovery of T-cell diversity in the first year after transplantation, followed by a faster diversification and by a very late reduction of T-cell heterogeneity only in the subgroup which had not undergone ablative conditioning; in contrast, those without GvHD showed a slower but constant and longer-lasting diversification of the T-cell repertoire throughout the follow-up.

## 4. Discussion

Here, we report a detailed analysis of the kinetics of new B- and T-lymphocyte production in 102 long-term followed-up children receiving HSCT as treatment for SCID and PID, compared to 22 children with SCID or PID that did not undergo transplantation, who were chosen as a control group. In HSCT-SCID patient, the pattern of KREC and TREC posttransplantation release was not different from that found in HSCT-PID, although the observed mean frequency of HSCT-SCID patients having KRECs and TRECs above the cut-off was lower, in particular one year after transplantation. In both groups the frequency of patients with KRECs above the cut-off remained high also at more than 10 years after transplantation. This similar trend of immune repopulation in SCID and PID transplanted patients is likely due to the fact that few SCID patients received unconditioned or nonmyeloablative transplants. Indeed, data collected retrospectively from 240 infants with SCID, who had received transplants from donors other than matched siblings at 25 centers during a 10-year period, indicated that reduced-intensity or myeloablative conditioning were both associated with improved T-cell counts and more consistent B-cell function [20].

In both groups of transplanted patients, the frequency of patients with TRECs above the cut-off slightly decreased over time. The time-related reduction of newly produced T cells appears to be more marked in HSCT-PID, but, considering that the frequency of patients with normal TRECs was higher in the samples obtained in the previous years, this frequency was still higher than in HSCT-SCID at the last point of the follow-up. A clear reduction of newly produced T cells was observed in the group of nontransplanted patients older than 10 years, and this is probably due to both age- and disease-related thymic involution [14, 21].

An efficient thymic output is the most important requirement for the generation of a broad array of different T cells able to respond to a large range of pathogens encountered by the host [10, 22]. Accordingly, we found that a constantly high frequency of patients (>50%) with a normal TREC production was paralleled by a rather conserved diversity of T-cell repertoire, which remained stable after the initial increase occurring 1 year after transplantation. Therefore, the slight decrease in the frequency of patients with TRECs above the cut-off observed more than 10 years after HSCT was likely due to a reduction in thymic output not sufficient,* per se*, to affect the recovered repertoire diversity. In this context, our statistical model predicts not only that a normal TREC production increases the probability of having an unrestricted repertoire, but also that a proportion of patients who developed GvHD have a distinct pattern of recovery of T-cell repertoire diversity, which is slower only in the first year after transplantation. This is not surprising because acute GVHD was shown to disrupt thymic epithelium reversibly [22]; in this context, our results extend and complement those by Clave et al. [22], who observed a delayed but fully reversible thymic function and repertoire restriction in young patients with acute GvHD in the first year following HSCT. Another factor possibly contributing to a slow repertoire diversification could be the immune suppressive therapy administered to patients with high grade acute GvHD; however, we could not include this element in the analysis due to missing information in older medical records. More importantly, we also found that, after the reprise of T-cell repertoire diversification, there is another trend towards a repertoire restriction in the very long term, which seems to be accounted for only by those who had not undergone an ablative conditioning. This peculiar kinetics is not completely surprising, however, because it is known that an ablative conditioning regimen is linked to a better T-cell reconstitution [20]. Finally, it is well known that also chronic GvHD induces a significant restriction of T-cell heterogeneity [22, 23]; however, in our study the number of patients with this condition was likely too small to have a significant impact on repertoire restriction.

Therefore, the present study, which analyzes the two most reliable markers of T-cell reconstitution in more than 1000 samples, may provide an improved explanation regarding the controversial issue of the duration of post-HSCT T-cell recovery in SCID patients. Indeed, while in the first studies T-cell recovery was evaluated through the development of normal T-cell counts and proliferative responses to phytohemagglutinin or recall antigen, more recently, researchers' attention has focused on the long-lasting thymic output resulting in a continuous production and development of a diverse T-cell repertoire. Accordingly, an initial report demonstrated that long-term transplanted SCID patients showed normal total T-cell counts and responses to mitogens [24] but, later, the same group found that T-cell immunity can be impaired later in life because of long-term graft failure or because of an increased rate of thymus impairment, which may be unable to sustain a sufficient T-cell output [7, 25]. Low TREC number and the T-cell repertoire oligoclonality were considered to derive from either loss of T-cell progenitors in the absence of donor stem cell engraftment or from thymic dysfunction caused by damage from infections, chemotherapy, or GvHD. However, another study found that in patients with a decreased long-lasting T-cell reconstitution, T-cell recovery appeared to be already poor early after HSCT, indicating that long-term immune failure was not caused by accelerated loss of thymic output or long-term graft failure, but resulted from poor early grafting [9]. A later investigation did not show a decline of CD3^+^ and CD4^+^ T cells 10 years and more after HSCT, suggesting that there was no long-term decrease of T-cell immunity [26]. This still unsettled issue can most likely derive from the present lack of longitudinal studies performed in a large number of long-term transplanted SCID patients analyzed at several time points. For instance, Buckley et al. [24] studied 89 SCID patients treated with HSCT up to 17 years earlier (median follow-up of 5.6 years), but CD3^+^ cell count and proliferative response were measured at two time points only, one before and one after transplantation. Patel et al. [25] evaluated the T-cell function at various times after HSCT in 73 SCID infants, but using only a single point from an individual patient in any given period, and Sarzotti et al. [7] quantified the thymic output and T-cell diversity in 16 children studied 10 years after HSCT. In a previous work they investigated the long-term immune reconstitution and clinical outcome in 40 patients with SCID that survived for up to 11 years after HSCT, but they reported the level of TRECs and the extent of T-cell heterogeneity only at the last point of follow-up [27]. Borghans et al. [9] analysed T-cell reconstitution in 35 children with SCID with a median follow-up of 12 years at two time points (short- and long-term follow-up), while Neven et al. [26] analysed CD3^+^ and CD4^+^ cells, and T-cell functions at four time points in 32 SCID patients with a follow-up from 5 to 10 years and in 35 patients with a follow-up >15 years. Furthermore, to our knowledge, only a study [28] has compared a group of 25 HSCT-SCID patients with 11 patients with other PID, but it was performed in short-term followed up patients (34.5 months versus 24 months) and thymic output was assessed only on a small subset of patients. Finally, no data are published reporting the measurement of thymic output and T-cell repertoire in a large number of samples. The present study was indeed performed to overcome the above limitations. To this aim, we chose a statistical method allowing us to utilize all available samples, regardless of total follow-up duration or interval between visits. In particular, our models could fit rather well the increasing-decreasing shape of the observed immune parameter change. However, one limitation is that, due to sample size restrictions (in particular if considering the no-HSCT group), a subgroup analysis could not be performed to compare patients with different causes of immune deficiency known to lead to a less likely immune recovery (e.g., KREC recovery in X-SCID versus other SCID).

Post-HSCT recovery of B cells has been so far less extensively investigated than T-cell reconstitution, but a recent review indicated that in SCID patients the qualitative restoration of B-cell function varies from 25% to 80% [29]. It is well known, indeed, that correction of B-cell function has been more problematic than that of T cell, thus requiring immunoglobulin infusions to prevent infections [24]. For instance, pretransplant conditioning of infants with SCID who do not have a matched sibling donor does not always result in B-cell function recovery [29], but immunoglobulin replacement tends to resolve B-cell dysfunction over time in some patients [26]. Several other factors have been shown to be associated with B-cell recovery, including myeloid donor engraftment, which was identified as the main predictor of recovery of B-cell function and T-cell reconstitution with stable thymic output [26, 30]. The kinetic of newly produced B-cells release from bone marrow was studied only in a low number of transplanted patients or in samples obtained early after HSCT [11, 12, 15, 31, 32]. This study found that, in SCID patients, KRECs had been constantly high since 2 years after HSCT, and therefore a defective B lymphocyte production should not be included among the causes of B-cell dysfunction after transplantation. While increasing data confirm that rise of KRECs is associated with enhanced B-cell neogenesis [11, 12, 15, 16, 18, 31–33], much less is known regarding KREC-associated humoral immune reconstitution after HSCT. However, it has been shown that transplanted children with low KRECs also have constantly low levels of one or more immunoglobulin subclasses [11], that allo-HSCT adult acute leukemic patients with late onset of KREC repopulation [32] also have an impaired antibody-mediated immune response, and that, on the contrary, the positivity for KRECs one month after transplantation is associated with decreased infectious episodes [34]. Therefore, taken together, these features hint a likely relationship between KREC recovery and B-cell immune function restoration. Nevertheless, we were not able to find a link between the probability of KREC reconstitution and factors like the presence or type of conditioning, which have been shown to affect the recovery of B-cell function after transplantation [35]. One explanation maybe, again, lack of statistical power, due to the low proportion of patients not receiving any conditioning. For this reason, we cannot exclude a true effect for these and other factors that were not found associated with the outcome of interests, even though they usually are in other studies, such as, for example, donor type [20] or dose of stem cells. All these limitations should be kept well in mind when dealing with our multivariable analysis conclusions.

## 5. Conclusions

The present data may provide the experimental ground to address the long-standing controversy regarding the duration of T-cell immune reconstitution in HSCT-SCID patients and demonstrate that posttransplantation long-term B- and T-cell reconstitution may be equally achieved in children with SCID and other PID as well.

## Supplementary Material

Supplementary Figure 1: Histograms reporting the frequency distributions of: A) the number of samples per patient; B) samples belonging to transplanted and non transplanted patients in each age-class; C) patients in the time-classes corresponding to the first and last sampling; C) patients with a follow-up duration comprised into each time-class.
Supplementary Figure 2: Adjusted predicted probabilities of having KRECs over the cut-off, TRECs over the cut-off, an unrestricted repertoire calculated at several representative time-points (corresponding approximately to those depicted in Fig. 1 and 2) in HSCT-SCID vs HSCT-PID patients. Error bars represent the 95% confidence interval.
Supplementary Figure 3: Adjusted predicted probabilities of having KRECs over the cut-off, TRECs over the cut-off, an unrestricted repertoire at several representative time-points (corresponding approximately to those depicted in Fig. 1 and 2) in HSCT vs No-HSCT-PID patients. Error bars represent the 95% confidence interval.
Supplementary Figure 4: Adjusted predicted probabilities of having an unrestricted repertoire according to the model #2 reported in Table 4, to show the variable effects of GvHD and ablative conditioning during the follow-up. Probabilities were calculated at representative time-points showing the variable patterns of change over time. Error bars represent the 95% confidence interval.

## Figures and Tables

**Table 1:** Characteristics of the patients included in the study and of the transplants.

	HSCT-		No-
	SCID	PID	HSCT
Number	54	48	22
Gender			
Male/female	31/23	35/13	18/4
Type of immunodeficiency/molecular defects			
IL-2G gene mutations	12		
JAK-3 gene mutations	11		
RAG1 and RAG2 mutations	7		
Artemis gene mutations	5		
IL-7R gene mutations	3		
ADA deficiency	6		6
Reticular dysgenesis	3		
Unknown molecular cause	7		
Wiskott-Aldrich syndrome		15	2
Omenn's syndrome for RAG1, RAG2, and RMRP mutations		12	
CD40L gene mutations		3	
IPEX for FOXP3 mutations		2	
FHL syndrome for PRF1 gene mutations		2	
LAD-1 for ITGB2 gene mutations		2	
Osteopetrosis for TINF2 gene mutations		2	
CHH for RMRP gene mutation		1	1
Kostmann disease for HAX1 gene mutation		1	
PNP deficiency		1	
Winget helix deficiency for FOXN1 gene mutation		1	
CID		6	4
XLT			8
XLA			1
Age at the transplant			
Mean	10	25	—
Donor type			
MSD	12	10	—
MMRD	22	8	—
MUD	20	30	—
T-depletion	23	7	—
Dose of stem cells (10^6^/Kg)			
Mean	15.9	9.8	—
Standard deviation	14.8	7.1	—
Conditioning			
None	11	3	—
Nonablative	5	1	—
Ablative	38	44	—
Use of ATG or other mAbs	19	29	—
Acute GvHD			
I	17	16	—
II	5	14	—
III-IV	7	2	—
Chronic GvHD	4	4	—
Follow-up duration^*^			
Total	95	97	104
Between samples	12	14	15

ADA: adenosine deaminase; Artemis: DNA cross-link repair 1C gene; ATG: rabbit thymoglobulin; CD40 L: CD40 ligand; CHH: cartilage hair hypoplasia; CID: combined immunodeficiency; FHL: familial hemophagocytic lymphohistiocytosis; FOXN1: forkhead box N1 transcription factor; FOXP3: forkhead box P3; GvHD: graft-versus-host disease; HSCT: hematopoietic stem cell transplantation; IL2RG: interleukin 2 receptor, gamma gene; IL7R: interleukin 7 receptor; IPEX: immune dysregulation, polyendocrinopathy, and enteropathy; ITGB2: integrin beta 2; JAK3: Janus kinase 3; LAD-1: leukocyte adhesion deficiency type 1; mAbs: monoclonal antibodies; MMRD: mismatched related family donor; MSD: HLA-matched sibling donor; MUD: unrelated donor; PID: primary immunodeficiency; PRF: perforin 1 (pore forming protein); RAG: recombination activating genes; RMRP: RNA component of mitochondrial RNA processing endoribonuclease; PNP: purine nucleoside phosphorylase; SCID: severe combined immunodeficiency; TINF2: TRF1-interacting nuclear factor 2; XLA: X-linked agammaglobulinemia; XLT: X-linked thrombocytopenia. ^*^Mean (months).

**Table 2:** Multivariable logistic models investigating differences in immune reconstitution between HSCT-SCID and -PID patients.

Outcome and contributing factors	OR	SE	*P*
Probability of having KRECs over the cut-off			
PID	1	—	—
SCID	0.2300	0.2421	0.1630
Age at transplantation	1.0150	0.0178	0.3940
Time^a^	1.0738	0.0167	0.0000
Time × time^b^	0.9998	0.0001	0.0000
Probability of having TRECs over the cut-off			
PID	1	—	—
SCID	0.4842	0.3562	0.3240
Age at transplantation	0.9942	0.0129	0.6550
Time	1.0420	0.0083	0.0000
Time × time^b^	0.9997	0.0000	0.0000
Probability of having an unrestricted repertoire			
PID	1	—	—
SCID	0.5076	0.1982	0.0820
Age at transplantation	0.9940	0.0069	0.3870
Time	1.0313	0.0058	0.0000
Time × time^b^	0.9999	0.0000	0.0000

OR: odds ratio; SE: standard error.

HSCT: hematopoietic stem cell transplantation; KRECs: K-deleting recombination excision circles; PID: primary immunodeficiency; SCID: severe combined immunodeficiency; TRECs: T-cell receptor excision circles.

^a^Time in months since transplantation; it represents the slope of the regression line (see Supplementary Figure  2); ^b^this factor represents the quadratic term needed to model the curved shape of time patterns, that is, the average monthly rate of change of the slope of the regression line.

**Table 3:** Multivariable logistic models investigating differences in immune reconstitution between HSCT and no-HSCT patients.

Outcome and contributing factors	OR	SE	*P*
Probability of having KRECs over the cut-off			
No-HSCT	1	—	—
HSCT	0.0111	0.0251	0.0470
Time^a^	0.9879	0.0276	0.6640
Time × time^b^	1.0001	0.0001	0.5150
HSCT × time	1.1002	0.0377	0.0050
HSCT × time × time^c^	0.9997	0.0001	0.0060
Probability of having TRECs over the cut-off			
No-HSCT	1	—	—
HSCT	0.2980	0.4389	0.4110
Time	0.9775	0.0209	0.2870
Time × time^b^	0.9999	0.0001	0.5120
HSCT × time	1.0608	0.0246	0.0110
HSCT × time × time^c^	0.9998	0.0001	0.0770
Probability of having an unrestricted repertoire			
No-HSCT	1	—	—
HSCT	3.3335	1.6877	0.0170
Time^a^	1.0231	0.0056	0.0000
Time × time^b^	0.9999	0.0000	0.0190
HSCT × time	n/i	—	—
HSCT × time × time^c^	n/i	—	—

OR: odds ratio; SE: standard error.

HSCT: hematopoietic stem cell transplantation; KRECs: K-deleting recombination excision circles; TRECs: T-cell receptor excision circles.

n/i: term not included in the model.

^a^Age in months at the moment of sampling; it represents the slope of the regression line (see Supplementary Figure  3); ^b^this interaction represents the quadratic term needed to model the curved shape of time patterns, that is, the average monthly rate of change of the slope of the regression line. ^c^This interaction term represents the effect of HSCT on the rate of change of the slope.

**Table 4:** Multivariable logistic models investigating factors affecting the probability of having an unrestricted repertoire in HSCT patients.

Outcome and contributing factors	OR	SE	*P*
Probability of having an unrestricted repertoire			
Model 1			
TRECs under the cut-off	1	—	—
TRECs over the cut-off	3.2391	0.8649	0.0000
GvHD × time^a^	1.0403	0.0119	0.0010
GvHD × time × time^b^	0.9998	0.0001	0.0030
Model 2			
TRECs under the cut-off	1	—	—
TRECs over the cut-off	2.8469	0.7616	0.0000
GvHD × time^a^	1.1050	0.0354	0.0020
GvHD × time × time^b^	0.9994	0.0354	0.0050
Ablative conditioning × GvHD × time^c^	0.9305	0.0329	0.0420
Ablative conditioning × GvHD × time × time^d^	1.0005	0.0002	0.0260

OR: odds ratio; SE: standard error.

Two models were fitted; reported here are only the significant factors and interactions; predictions of model 2 are depicted in Supplementary Figure  4.

GvHD: graft-versus-host disease.

TRECs: T-cell receptor excision circles.

^a^Time expressed in months since transplantation; the interaction represents the effect of GvHD on the slope of the regression line (see Supplementary Figure  4); ^b^this quadratic interaction represents the effect of GvHD on the rate of change of the slope, that is, the curvature of the regression line; ^c,d^these interactions represent the change of the effect of GvHD on the slope due to the presence or absence of ablative conditioning.
