# Supplementary material for: Long-Lasting Production of New T and B Cells and T-Cell Repertoire Diversity in Patients with Primary Immunodeficiency Who Had Undergone Stem Cell Transplantation: A Single-Centre Experience
Source: J Immunol Res. 2014 Dec 1;2014:240453. doi: 10.1155/2014/240453 (PMC4270024; doi:10.1155/2014/240453)
Supplement: Supplementary file 1 — Supplementary Figure 1: Histograms reporting the frequency distributions of: A) the number of samples per patient; B) samples belonging to transplanted and non transplanted patients in each age-class; C) patients in the time-classes corresponding to the first and last sampling; C) patients with a follow-up duration comprised into each time-class. Supplementary Figure 2: Adjusted predicted probabilities of having KRECs over the cut-off, TRECs over the cut-off, an unrestricted repertoire calculated at several representative time-points (corresponding approximately to those depicted in Fig. 1 and 2) in HSCT-SCID vs HSCT-PID patients. Error bars represent the 95% confidence interval. Supplementary Figure 3: Adjusted predicted probabilities of having KRECs over the cut-off, TRECs over the cut-off, an unrestricted repertoire at several representative time-points (corresponding approximately to those depicted in Fig. 1 and 2) in HSCT vs No-HSCT-PID patients. Error bars represent the 95% confidence interval. Supplementary Figure 4: Adjusted predicted probabilities of having an unrestricted repertoire according to the model #2 reported in Table 4, to show the variable effects of GvHD and ablative conditioning during the follow-up. Probabilities were calculated at representative time-points showing the variable patterns of change over time. Error bars represent the 95% confidence interval. [file 240453.f1.zip › Supplementary_Fig_1_CDI_1097407.pptx]

## Slide 1
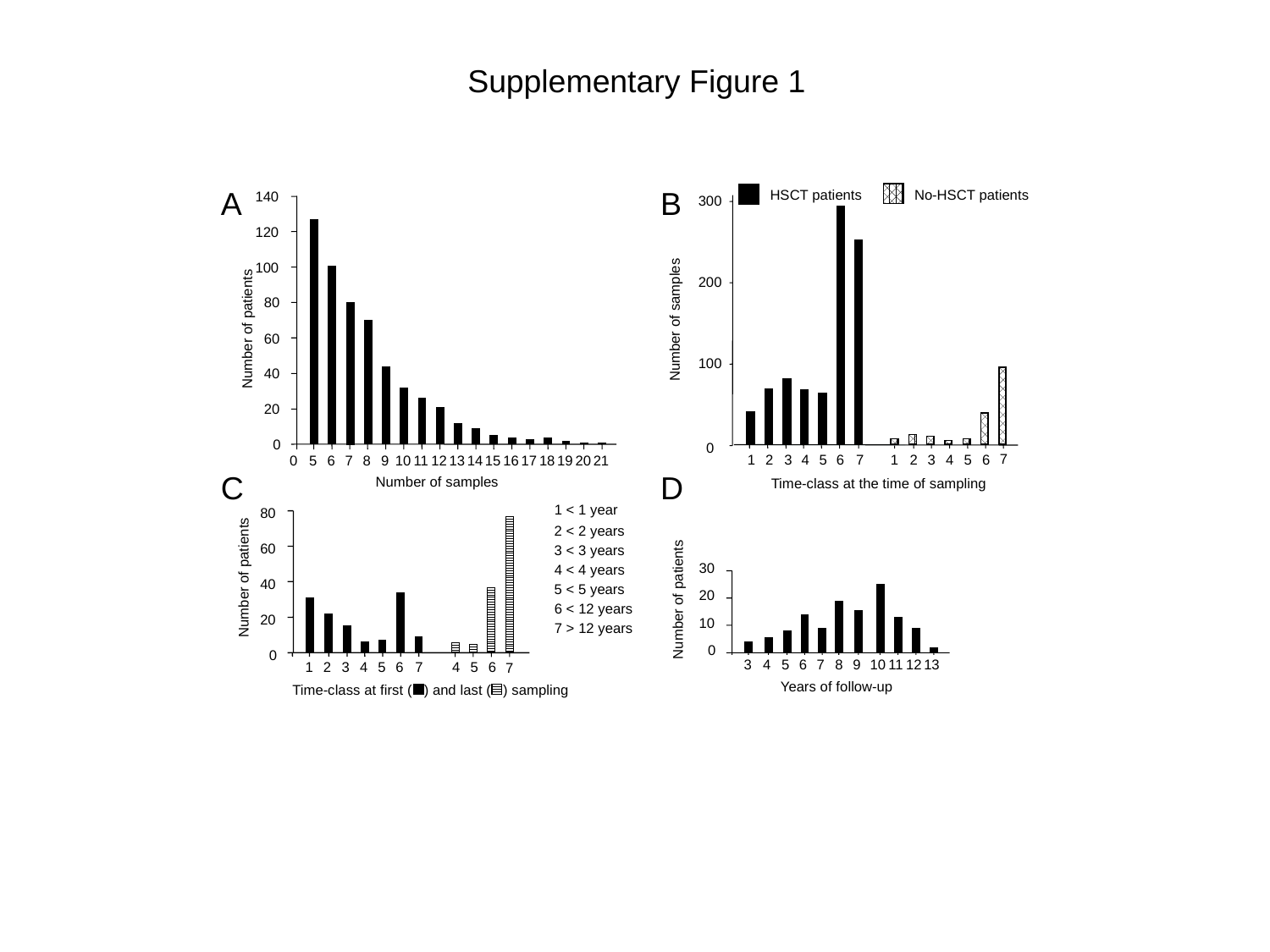

Supplementary Figure 1
A
B
HSCT patients
140
300
6
120
7
100
200
80
Number of samples
Number of patients
60
100
40
3
2
4
5
20
1
0
0
7
1
2
3
4
5
6
0
5
6
7
8
9
10
11
12
13
14
15
16
17
18
19
20
21
C
D
Number of samples
Time-class at the time of sampling
1 < 1 year
80
2 < 2 years
30
10
20
Number of patients
8
9
10
6
11
7
12
5
4
0
3
13
Years of follow-up
60
3 < 3 years
4 < 4 years
Number of patients
40
5 < 5 years
6 < 12 years
20
7 > 12 years
0
1
2
3
4
5
6
7
4
5
6
7
Time-class at first ( ) and last ( ) sampling
No-HSCT patients
