# Supplementary material for: Long-Lasting Production of New T and B Cells and T-Cell Repertoire Diversity in Patients with Primary Immunodeficiency Who Had Undergone Stem Cell Transplantation: A Single-Centre Experience
Source: J Immunol Res. 2014 Dec 1;2014:240453. doi: 10.1155/2014/240453 (PMC4270024; doi:10.1155/2014/240453)
Supplement: Supplementary file 1 — Supplementary Figure 1: Histograms reporting the frequency distributions of: A) the number of samples per patient; B) samples belonging to transplanted and non transplanted patients in each age-class; C) patients in the time-classes corresponding to the first and last sampling; C) patients with a follow-up duration comprised into each time-class. Supplementary Figure 2: Adjusted predicted probabilities of having KRECs over the cut-off, TRECs over the cut-off, an unrestricted repertoire calculated at several representative time-points (corresponding approximately to those depicted in Fig. 1 and 2) in HSCT-SCID vs HSCT-PID patients. Error bars represent the 95% confidence interval. Supplementary Figure 3: Adjusted predicted probabilities of having KRECs over the cut-off, TRECs over the cut-off, an unrestricted repertoire at several representative time-points (corresponding approximately to those depicted in Fig. 1 and 2) in HSCT vs No-HSCT-PID patients. Error bars represent the 95% confidence interval. Supplementary Figure 4: Adjusted predicted probabilities of having an unrestricted repertoire according to the model #2 reported in Table 4, to show the variable effects of GvHD and ablative conditioning during the follow-up. Probabilities were calculated at representative time-points showing the variable patterns of change over time. Error bars represent the 95% confidence interval. [file 240453.f1.zip › Supplementary_Fig_4_CDI_1097410.pptx]

## Slide 1
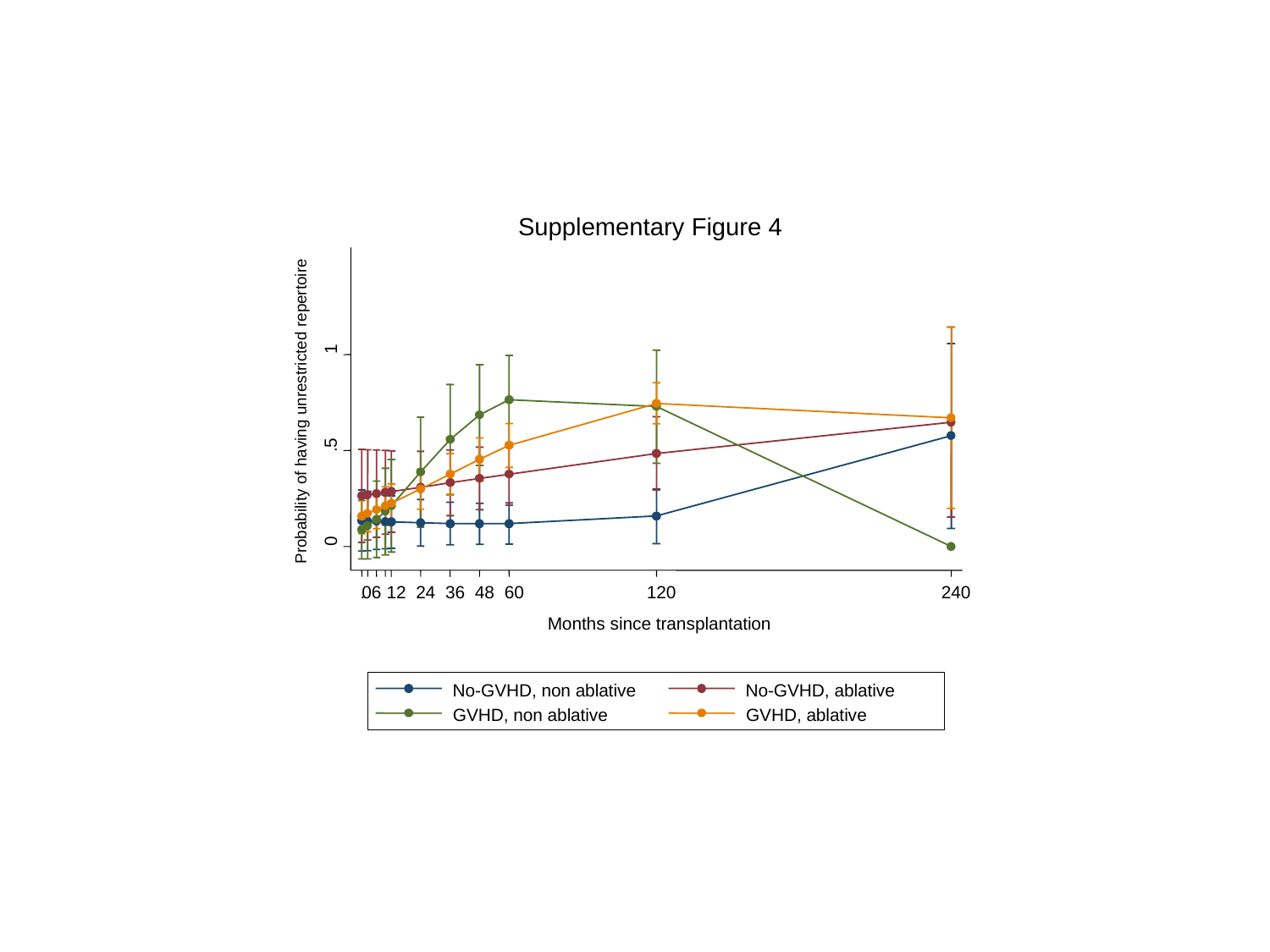

Supplementary Figure 4
1
Probability of having unrestricted repertoire
.5
0
.
6
12
24
36
48
60
120
240
0
Months since transplantation
No-GVHD, non ablative
No-GVHD, ablative
GVHD, non ablative
GVHD, ablative
